# Impact of dispersion time interval and particle size on release profiles of propranolol HCl and carbamazepines from microparticle blends system

**DOI:** 10.1038/s41598-022-14678-w

**Published:** 2022-06-20

**Authors:** Muhaimin Muhaimin, Anis Yohana Chaerunisaa, Roland Bodmeier

**Affiliations:** 1 Faculty of Pharmacy, Universitas Padjadjaran, Jl. Raya Bandung-Sumedang Km 21, Jatinangor, 45363 Indonesia; 2 College of Pharmacy, Freie Universität Berlin, Kelchstr. 31, Berlin, 12169 Germany

**Keywords:** Drug discovery, Chemistry, Materials science

## Abstract

The objective of this study was to investigate the effect of dispersion time interval (DTI) on physicochemical properties of drug following the incorporation of propranolol HCl (Pro) and carbamazepine (CBZ) within ethyl cellulose (EC) microparticle blends using solvent evaporation method. The first Pro emulsion and second CBZ oil phase were dispersed in an external aqueous phase, with DTI of 0 and 60 min. The morphology of microparticle blends were characterized by SEM. The particle size mean of the emulsion droplets/hardened microparticles were monitored by FBRM. Encapsulation efficiency (EE) and in vitro drug release were also investigated. The resulting microparticle blends were spherical and formed two populations. The particle size mean of microparticle blends ranged from 113.27 µm to 122.42 µm. The EE was 77.28% to 78.64% for Pro and 96.48% to 98.64% for CBZ. FBRM studies showed that the size of microparticle blend prepared as W/O/W (Pro) and O/W (CBZ) system with DTI of 60 min and stirring time 4 h were larger than those prepared with DTI of 0 min. In vitro drug release studies after 28 days that revealed the CBZ release (58.72%) was faster than Pro release (43.16%). Investigation on surface morphology by SEM showed that the second drug CBZ which added as the oil phase in the W/O/W emulsion system had blocked the pores on the surface Pro microparticles prepared from the first primary emulsion, therefore affecting the drug release. This blocking effects of second drug (CBZ) on first emulsion microparticles (Pro) depended on the DTI. This phenomenon is only applicable if the first primary emulsion is W/O/W system.

## Introduction

Microparticles are widely used in different applications such as the controlled release of drugs, cosmetics and chemical reagents. Several methods are potentially useful for the preparation of microparticles in the field of controlled drug delivery. The solvent evaporation technique is one of the most frequent ways for generating microparticles^1–9^. Controlling the microparticle preparation processes is critical for achieving the required mean size, size distribution and shape of microparticles. When choosing emulsion phases for a microparticles preparation process, the solubility of the active drug are critical features to consider. Many types of drugs including small molecules or large molecule such as proteins and nucleic acids can be encapsulated in microparticles^6, 9–15^. Simple or multiple emulsion procedures such as oil-in-water (O/W) or water-in-oil-in-water (W/O/W) are utilized based on the drug's solubility^16–18^. In the encapsulation and release of pharmaceuticals the process of microparticle production is a determining factor^19–23^. Furthermore, many parameters such as the type of polymer, polymer molecular weight, copolymer composition, nature of excipients used in the microparticle formulation (e.g., for drug stability), porosity and microparticle size can all have a significant impact on distribution rates^24–30^.

Polymers have been utilized extensively to modulate the rate of drug release from formulations. Polymers have the ability to bind solid dosage form particles. Taste masking, controlled release (e.g., prolonged, pulsatile, and targeted), better stability, and higher bioavailability are all common uses for pharmaceutical polymers^31–35^. Non-biodegradable polymers with strong biocompatibility like ethyl cellulose are also utilized as drug carriers (degradable but non biodegradable). Non-ionic ethyl ether of cellulose (EC) is a cellulose derivative where such part of the hydroxyl groups on the repeating anhydroglucose units are converted into ethyl ether groups. Due to its versatile properties such as being water insoluble but soluble in many organic solvents such as alcohol, ether, ketone and ester; being biocompatible and suitable with many celluloses, resins and almost all plasticizers; being stable against light, heat, oxygen and wetness and chemicals; and being non-toxic. EC has been widely utilised in microencapsulation^36^. EC is used to microencapsulate drugs in order to protect them against active interactions, hydrolysis and oxidation. It is indeed also used as a matrix and/or coating agent to provide long-term release properties.

In the majority of trials so far, only one medication was captured at a time within controlled release microparticles. Only a few efforts at co-encapsulating two medications have been performed, especially if the latter has a markedly altered solubility behavior. Pérez et al. (2000) used solvent evaporation procedures to insert a lipophilic and a hydrophilic drug into biodegradable poly(ε-caprolactone)-based microparticles^27^. Pérez et al. (2003) effectively included the hydrophilic medicine propranolol HCl and/or the lipophilic drug nifedipine individually and simultaneously within non-degradable, ammonio methacrylate copolymer (Eudragit RS:RL 4:1 blends)^16^. These microparticles were made using the solvent evaporation methods of oil-in-water (O/W) and water-in-oil-in-water (W/O/W). Nippe and General (2012) established lipophilic steroidal drugs ethinyl estradiol and drospirenone poly(lactic-co-glycolic acid) (PLGA) microparticles, whereas they developed a combination of lipophilic steroidal drugs ethinyl estradiol and drospirenone poly(lactic-co-glycolic acid) (PLGA) microparticles^37^. Combination products often known as fixed dose combos are single-dose formulations of two or more active medications. These product provide the advantages of combination therapy while useful to improve adherence and can simplify procurement, storage and distribution of medicines. Fixed dose combination drugs are an important approach to addressing the management of both chronic and acute diseases.

There haven’t been any reports of microparticle blends comprising two drug with different solubilities. The solvent evaporation approach was utilized to introduce a lipophilic and a hydrophilic drug into ethyl cellulose-based microparticle blends in the current investigation. Model drug used in the experiment included the hydrophilic propranolol HCl and the lipophilic carbamazepine. The production of microparticle blends from oil-in-water (O/W) and water-in-oil-in-water (W/O/W) technologies requires accurate particle size analysis throughout the solvent evaporation process. FBRM may be utilized to give in situ/on-line particle characterisation in a variety of applications for more information regarding micropaticle blends creation during the solvent evaporation process^7, 8, 38–46^. The advantage of this method is that data is collected in real time and on-line to provide particle size data and population trends of particles in suspension, emulsion etc^7, 8, 38, 39, 41, 45–50^.

The purpose of this study was to investigate effect of dispersion time interval (DTI) and the formulation of second oil phase on EC-based microparticle blends contained drugs with different solubility (Pro and CBZ) which prepared by solvent evaporation method.

## Result and discussion

### Morphology and particle size/distribution of microparticle blends

**Figure 1:**
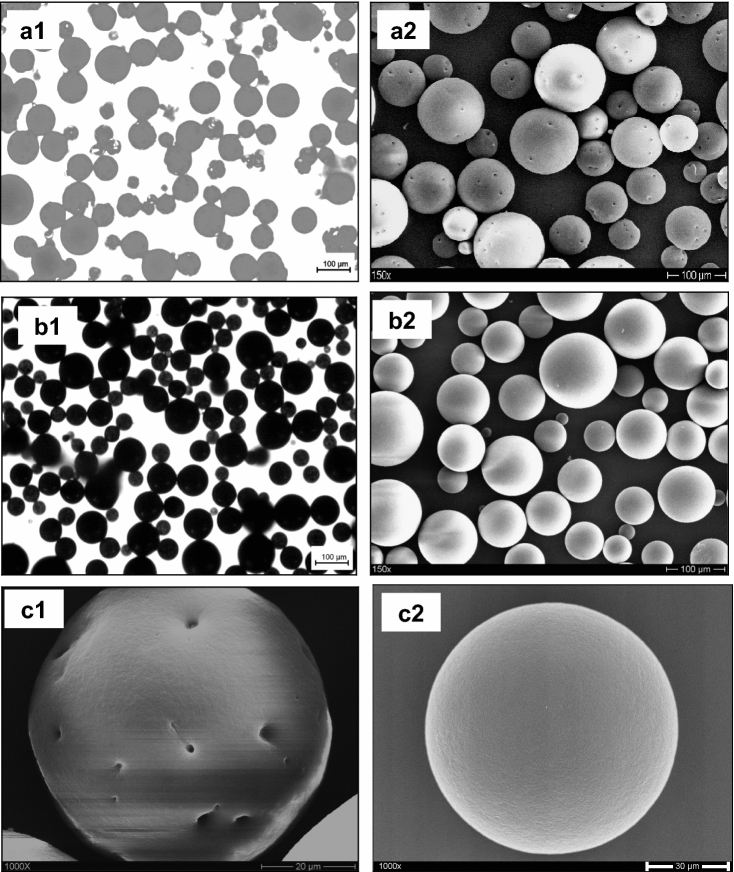
(1) Optical microscopy pictures [(**a1**) without dye; (**b1**) containing dye (black)]; (2 and 3) SEM pictures. [(**a2**) Pro (W/O/W); (**b2**) CBZ and dye (O/W) of ethyl cellulose microparticles]; and [(**c1**) Pro (W/O/W) of ethyl cellulose microparticles (1000X Magnification); (**c2**) CBZ (O/W) of ethyl cellulose microparticles (1000X Magnification)].

Microparticle blends were developed for this investigation used Pro and CBZ as model of drug with different solubilities. Scanning electron microscopy was used to examine the microparticles' surface morphology. Pro loaded microparticles (W/O/W) and CBZ loaded microparticles (O/W) exhibit spherical form, smooth surface (CBZ) and porous surface (Pro) according to SEM and optical microscopy images (Fig. 1). The Pro loaded microparticles (W/O/W) possessed several porous surface. It was caused by the inner aqueous phase. The aqueous droplets are precursors of pores and are the result of phase separation occurring in the organic phase during the hardening of the microparticles^2–5^. While the O/W emulsion system consists of an organic phase comprised of a volatile solvent with dissolved polymer and the drug to be encapsulated, emulsified in an aqueous phase containing a dissolved surfactant. For insoluble or poorly water-soluble drugs, the oil-in-water (O/W) method is frequently used. This method is the simplest and the other methods derive from this one^2–5^.

**Figure 2:**
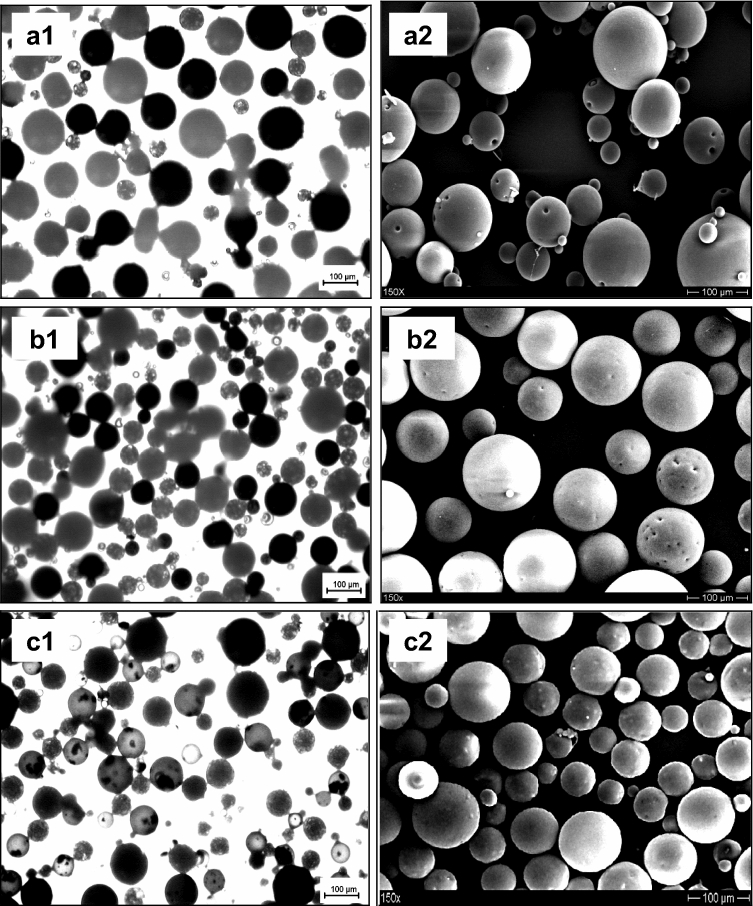
Optical microscopy pictures [**a1**, **b1** and **c1** containing dye (black)] (1) and SEM pictures (2) of ethyl cellulose microparticle blends with varying dispersion time interval and different primary emulsion. [(**a**) CBZ (O/W) as primary emulsion added by Propranolol (W/O/W), DTI of 60 min; (**b**) Pro (W/O) as primary emulsion added by CBZ (O/W), DTI of 0 min; (**c**) Pro (W/O) as primary emulsion added by CBZ (O/W), DTI of 60 min].

**Figure 3:**
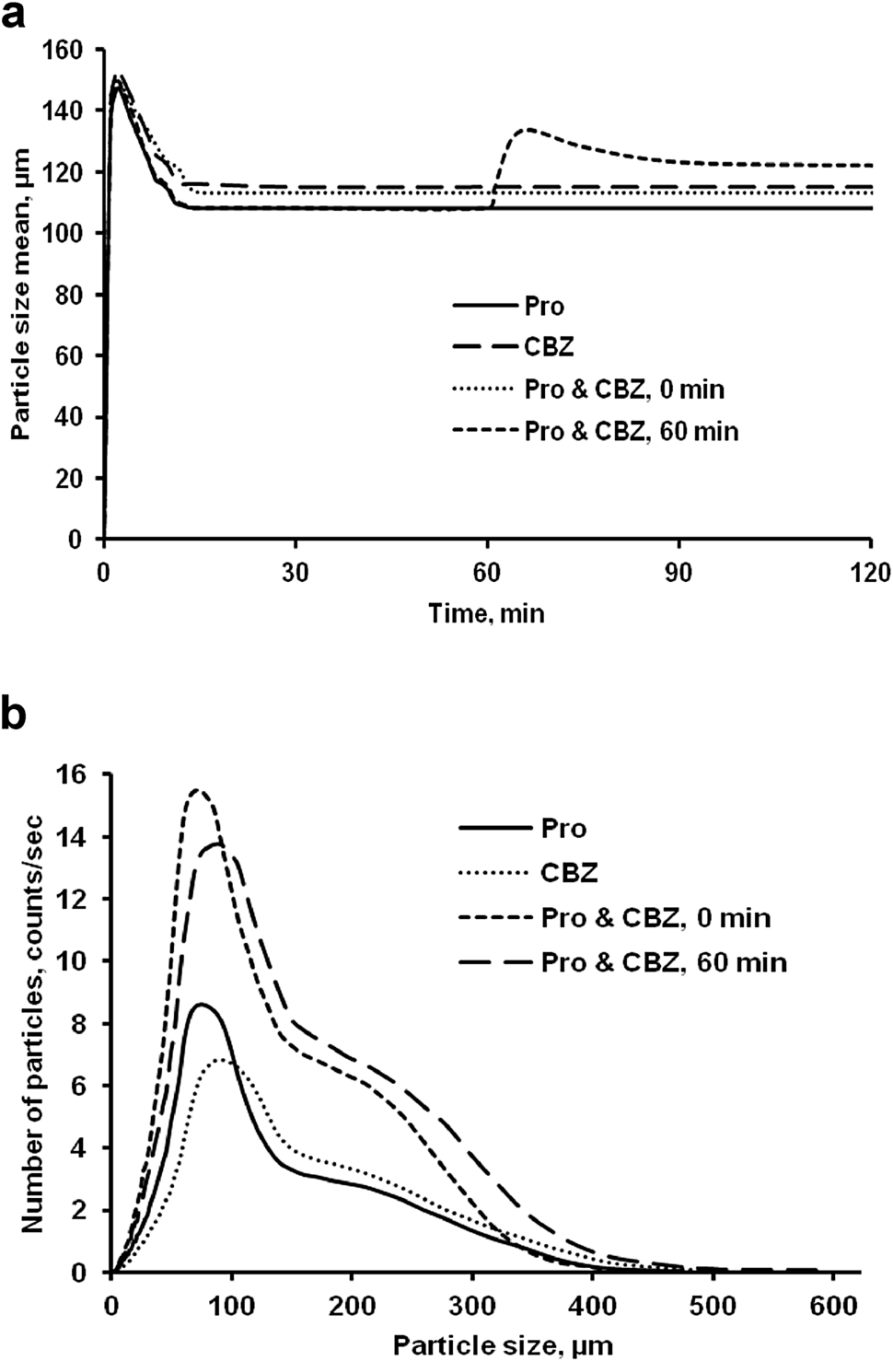
Particle size mean of ethyl cellulose based microparticle blends obtained by the FBRM method (before and after oil phase addition) during the solvent evaporation process (primary oil phase is added at time = 60 min) (**a**), and Particle size distribution obtained by the FBRM method for all batches of ethyl cellulose based microparticles blend (at 4 h stirring time) (**b**).

Surface analysis of drug-loaded microparticle blends generated by the WO/W (Pro) and O/W (CBZ) revealed that the microparticles were spherical and not aggregated (Fig. 2), with diameters ranging from 113.27 to 122.42 µm (Fig. 3a and Table 1). Size of microparticle blends was higher and different with other microparticle when DTI of 60 min with first primary emulsion (Pro) and second oil phase (CBZ) (*P* < 0.05) compared other microparticle (microparticle normal, microparticle blends (with DTI of 0 min) and microparticle blends (first primary oil phase (CBZ) and second primary emulsion (Pro), DTI of 60 min). The comparisons were made between different kind of microparticle using one way analysis of variance. *P* < 0.05 was considered as the significant level. Optical microscopy pictures (Fig. 2.a1,b1 and c1) revealed two populations of microparticles that consist of microparticles without dye (white) and containing dye (black).

**Table 1:** Formulation, drug entrapments and particle size mean of microparticles (whole size).

Drug		Dispersion time interval (minute)	Actual drug loading (%) (± SD)		Encapsulation efficiency (%) (± SD)		Particle size mean (µm) (± SD)
Emulsion 1	Emulsion 2		Pro	CBZ	Pro	CBZ	
Pro	–	–	9.62 (± 0.35)	–	76.96 (± 2.83)	–	108.38 (± 4.07)
CBZ	–	–	–	12.31 (± 0.08)	–	98.48 (± 0.65)	115.09 (± 5.12)
Pro	CBZ	0	9.78 (± 0.28)	12.15 (± 0.07)	78.24 (± 2.26)	97.20 (± 0.53)	113.27 (± 4.35)
Pro	CBZ	60	9.83 (± 0.27)	12.33 (± 0.04)	78.64 (± 2.13)	98.64 (± 0.32)	122.42 (± 6.04)
CBZ	Pro	60	9.66 (± 0.31)	12.06 (± 0.11)	77.28 (± 2.51)	96.48 (± 0.84)	117.35 (± 3.25)

Pro: consisted of propranolol HCl (DL 12.5%), ethyl cellulose and dichloromethane.

CBZ: consisted of carbamazepine (DL 12.5%), ethyl cellulose and dichloromethane.

Microparticle blends containing both propranolol HCl and carbamazepine, generated by the W/O/W (Pro) and O/W (CBZ) methods with DTI of 60 min appeared as two populations with smooth and rough surface (Fig. 2.c2). While microparticle blends which prepared with DTI of 0 min (Fig. 2.b2) produced microparticles with pores and smooth surface. This phenomenon was the same for preparing microparticle blends with emulsification stage, the first O/W (CBZ) and second W/O/W (Pro) with DTI of 60 min produced microparticles with pores and smooth surface (Fig. 2.a2). Micropores on the microparticles' surface indicated that the microparticles were Pro-loaded EC microparticles. There were no pores on the surface of the microparticles, indicating that the microparticles were CBZ-loaded EC microparticles.

The morphology and porosity of the microparticles were significantly impacted by the preparation procedures. The microparticles revealed a porous inner structure due to the inner aqueous phase in the W/O/W procedure. The aqueous droplets are precursors of pores and are the result of phase separation occurring in the organic phase during the hardening of the microparticles^2–4, 16, 27, 51–54^. A W/O/W multiple emulsion solvent evaporation method is mostly used for the encapsulation of water-soluble drug and therefore, was the method of choice for the water-soluble Pro drug. The W/O/W double emulsion method, the aqueous solution of hydrophilic drug is emulsified with organic phase (W/O emulsion), this emulsion is then dispersed into a second aqueous solution forming a second emulsion (W/O/W double emulsion). Highly porous microparticles were prepared by a double emulsion (W/O/W)^2, 4, 9^.

The size of microparticle blends generated by W/O/W (Pro) and O/W (CBZ) methods (with DTI of 60 min and stirring time 4 h) was larger than microparticle blends (with DTI of 0 min) and microparticles normal (Fig. 3). Before and after the primary oil phase was added to a single external aqueous phase, particle size mean/distribution was calculated (Fig. 3a,b). According to FBRM data, adding a second primary oil phase contained EC, carbamazepine and dichloromethane (with DTI of 60 min) contributed in particle size enhancement.

### Entrapment efficiency within microparticle blends

The encapsulation efficiency (EE) was about 77.28% to 78.64% for Pro and 96.48% to 98.64% for CBZ in microparticle blends containing different drugs (Table 1). In microparticles blends prepared by solvent evaporation method, the amount of drug entrapped in microparticle for Pro was lower and for CBZ was similar than the theoretical value. The percentage encapsulation efficiency was expressed by comparing the actual Pro and CBZ loading with the theoretical Pro and CBZ loading. In all formulations and experiments, the mean amount of drug entrapped was similar for Pro and also for CBZ (*P* < 0.05), each sample was assayed in triplicate. The comparisons were made between different preparations for the quality of encapsulation efficiency of Pro and also for CBZ using one way analysis of variance. *P* < 0.05 was considered as the significant level.

The varying solubilities of the drugs in the aqueous continuous phase employed for the two encapsulating procedures can explain the variation in the EE of the two drugs in the microparticle blends. The drug leaked into the continuous phase due to the high solubility of propranolol HCl in the external aqueous phase and its large volume compared to the internal aqueous phase (W/O/W method). This leakage is thought to occur mainly during the first minutes of emulsification because the polymer precipitates quickly thereby reducing leakage^16, 27, 52, 55, 56^. However, due to its hydrophilic nature, propranolol HCl tends to permeate through the polymeric matrix into the external aqueous phase once the polymer has precipitated. Furthermore, for the entrapment of ionizable drugs like Pro, the degree of ionization of the drug and the pH of the external aqueous phase are crucial^16, 27^. When the pH of the external phase is raised above the pKa of Pro, its solubility decreases and as a result that its trapping in microparticles increases.

### Release of propranolol HCl and carbamazepine from microparticle blends

**Figure 4:**
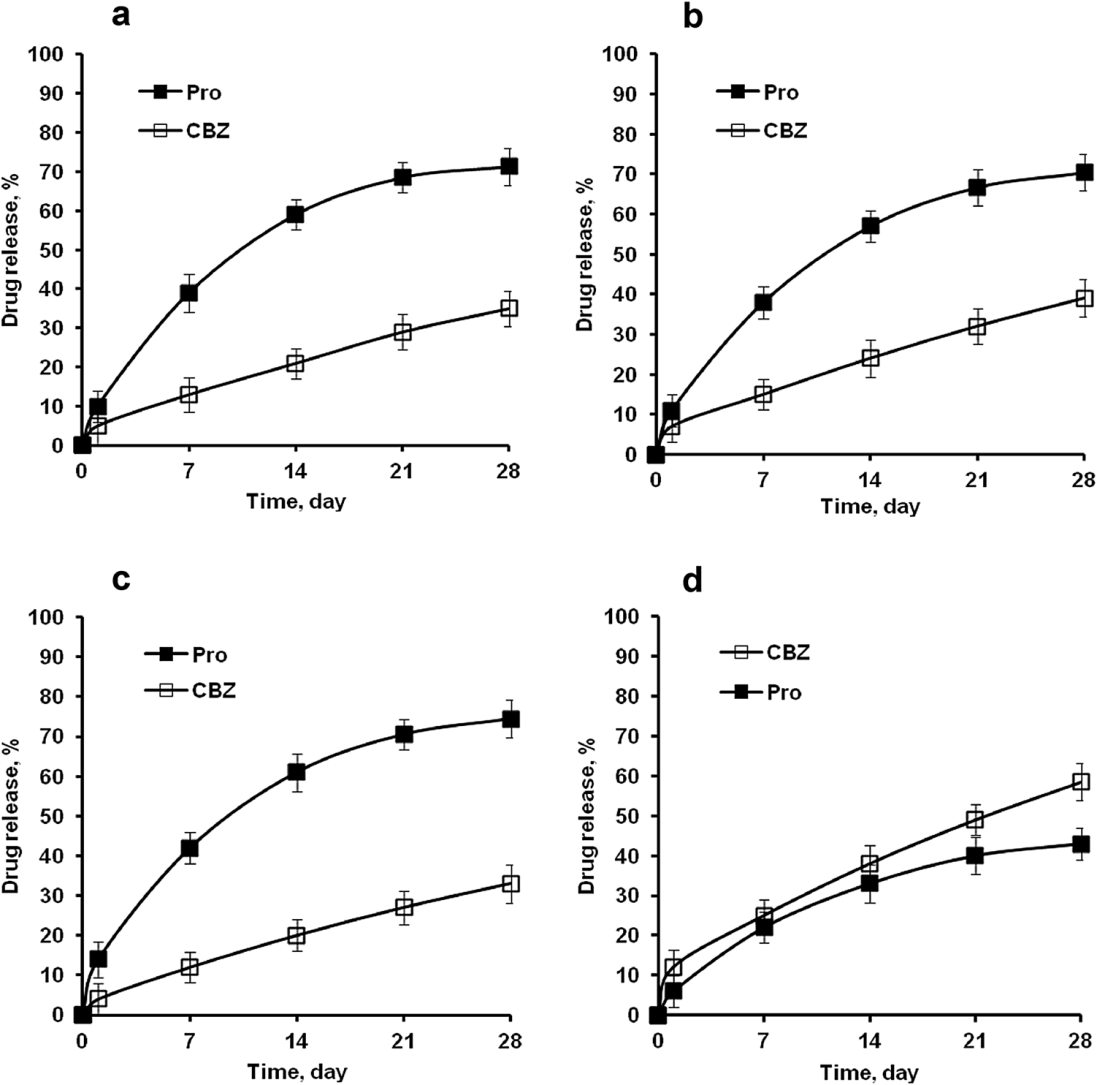
Effects of the dispersion time interval between primary emulsion and primary oil phase on propranolol HCl and carbamazepine release from ethyl cellulose microparticle blends (phosphate buffer, pH 7.4, 37 °C, 75 rpm). [**a**. Pro (W/O/W) [single drug], CBZ (O/W) [single drug]; **b**. Pro (W/O/W) and CBZ (O/W), DTI of 0 min; **c**. CBZ (O/W) and Pro (W/O/W), DTI of 60 min; d. Pro (W/O/W) and CBZ (O/W), DTI of 60 min].

Different release rates were observed for Pro and CBZ from EC microparticle blends in pH 7.4 phosphate buffer (Fig. 4).

The Pro release from microparticle normal, microparticle blends (with DTI of 0 min), microparticle blends (first primary oil phase (CBZ) and second primary emulsion (Pro), DTI of 60 min) were faster than CBZ release (Fig. 4a–c). Pro release (43.16%) was slower than CBZ release (58.72%) from EC microparticle blends (first primary emulsion (Pro) and second primary oil phase (CBZ), DTI of 60 min) (Fig. 4d). Figure 4 and Table 2 shows that the cumulative percent of Pro and CBZ released from each microparticle blends (the range of ADL Pro ≈ 8.59% to 8.64% and ADL CBZ ≈ 10.02% to 10.26%) at pH 7.4 after 28 days are in the range of 43.16% to 74.39% (Pro) and 33.05% to 58.72% (CBZ).

**Table 2:** Cumulative release of propranolol HCl and carbamazepine from EC microparticles (particle size: < 70 µm) in phosphate buffer (pH 7.4) after 28 days.

Drug		Dispersion time interval (minute)	Actual drug loading (%, w/w) (± SD)		Drug release (%) (± SD)	
Emulsion 1	Emulsion 2		Pro	CBZ	Pro	CBZ
Pro	–	–	8.62 (± 0.41)	–	71.32 (± 4.73)	–
CBZ	–	–	–	10.26 (± 0.14)	–	35.06 (± 4.51)
Pro	CBZ	0	8.59 (± 0.34)	10.08 (± 0.08)	70.44 (± 4.48)	39.18 (± 4.65)
Pro	CBZ	60	8.64 (± 0.36)	10.19 (± 0.06)	43.16 (± 4.06)	58.72 (± 4.55)
CBZ	Pro	60	8.61 (± 0.39)	10.02 (± 0.12)	74.39 (± 4.72)	33.05 (± 4.82)

Pro: consisted of propranolol HCl (DL 12.5%), ethyl cellulose and dichloromethane.

CBZ: consisted of carbamazepine (DL 12.5%), ethyl cellulose and dichloromethane.

The encapsulation efficiency of Pro and also for CBZ in all formulations and experiments of EC microparticles with particle size < 70 µm were determined (Table 2). Each sample was assayed in triplicate. The comparisons were made between different preparations for the quality of encapsulation efficiency of Pro and also for CBZ using one way analysis of variance with *P* < 0.05 was considered as the significant level. The results show the mean amount of drug entrapped was similar for Pro and also for CBZ (*P* < 0.05).

The amount of Pro and CBZ released were evaluated. Experiments were preformed in triplicate. Data are presented as average with the standard deviations. Comparisons were made between different preparation procedures for the quality of released of Pro and CBZ in vitro using one way analysis of variance. *P* < 0.05 was considered as the significant level. When DTI of 60 min for microparticle blends with first primary emulsion (Pro) and second oil phase (CBZ), the propranolol HCl release was slower than Carbamazepine release from microparticle blends compered other preparation procedures (*P* < 0.05). This may be due to second emulsion (CBZ) had blocked and coated pores on microparticle from first primary emulsion which contain Pro, resulting in no pores on surface and smaller pores inner structure of microparticle.

Particle size of microparticle blends influenced the rate of Pro and CBZ releases. Table 3 shows the mean diameters of five different size fractions of this microparticle, which range from 38.05 µm to 154.07 µm.

**Table 3:** Mean diameter and actual drug loading of different size fractions of propranolol HCl and carbamazepine from EC based microparticle blends with different dispersion time interval (theoretical drug loading = 12.5% w/w).

Size fraction (µm)	Mean diameter (µm) (± SD)		Actual drug loading (%, w/w) (± SD)			
			Propranolol HCl		Carbamazepine	
	0 min	60 min	0 min	60 min	0 min	60 min
< 41	38.05 (± 3.38)	39.04 (± 3.82)	6.86 (± 0.34)	6.91 (± 0.32)	8.79 (± 0.09)	8.83 (± 0.07)
41–70	63.17 (± 4.06)	68.11 (± 4.71)	8.94 (± 0.29)	9.12 (± 0.27)	10.45 (± 0.05)	10.56 (± 0.04)
71–100	92.88 (± 5.17)	97.24 (± 5.63)	10.11 (± 0.36)	10.22 (± 0.33)	11.35 (± 0.07)	11.43 (± 0.06)
101–160	147.28 (± 4.75)	154.07 (± 5.02)	11.27 (± 0.31)	11.35 (± 0.28)	12.41 (± 0.08)	12.47 (± 0.05)

**Figure 5:**
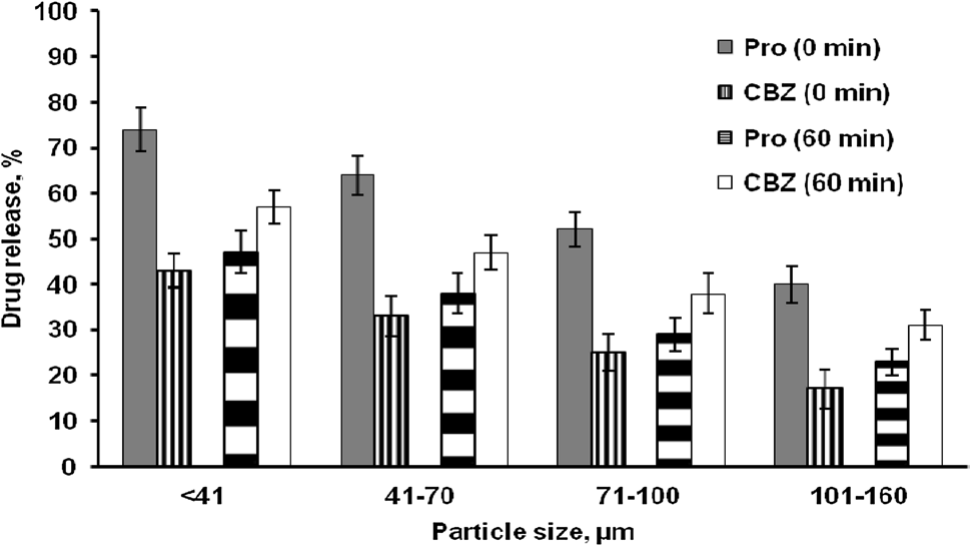
Effect of the size of ethyl cellulose based microparticle blends on propranolol HCl and carbamazepine release in phosphate buffer pH 7.4 (after 28 days).

The release of Pro and CBZ increase as particle size decreased (Fig. 5). Pro release was slower than that of CBZ for all size fractions as shown in Fig. 5 (with DTI of 60 min). In contrast, at DTI of 0 min the Pro release was faster than CBZ. Clearly, the size of the microparticles had effect on the rate of drug release which due to the particle's surface area-to-volume ratio rises as its size decreases. As a result, with a given rate of drug diffusion through microparticles, the rate of drug flux out of microparticles per mass of formulation will rise as particle size decreases. Furthermore, due to the shorter distance between the interface and the particle’s center, water penetration into smaller particles may be faster^18, 29, 53^. Diffusion is known to play a crucial role in the control of drug release from EC-based microparticles, hence a larger system is likely to result in lower relative release rates. It's because the diffusion channels have lengthened resulting in lower drug concentration gradients.

The resulting release rate(s) of the integrated drug(s) was/were determined to be regulated over at least 28 days in all situations. Additionally, CBZ release was generally slower than Pro, that can likely be due to CBZ's lower solubility in the release medium (0.2 mg/ml vs. 250 mg/ml), resulting in lower concentration gradients which are the driving factors for diffusion. Another explanation is that EC is insoluble in water and has a very low permeability. Since this formulation did not contain any channeling agents, formation of pores and cracks did not occur to facilitate drug release. Pro release remained slower than CBZ release when Pro loaded EC microparticles were combined with a second polymer organic formulation containing CBZ (with DTI of 60 min). The interaction of the second primary oil phase (CBZ) with hard particles from the first primary emulsion (Pro) could explain this behavior (Pro).

The integration of CBZ on the surface of Pro loaded microparticles may explain the increased carbamazepine release from microparticle blends with the W/O/W (Pro) and O/W (CBZ) (DTI of 60 min). When compared to microparticles generated using the W/O/W method, where Pro is either molecularly distributed or amorphous in the matrix, this may have reduced Pro migration to the surface of the microparticles and their leakage into the dissolution medium. Furthermore, the porous membrane found in microparticles generated using the W/O/W technique favored rapid release of the hydrophilic Pro. The hydrophobic character of CBZ and its extremely low water solubility may contribute to its inadequate release from microparticles.

On the contrary, the release of Pro was significantly slowed down in the case of the microparticle blends (DTI of 60 min) compared to that of the microparticles normal. Only 43.16% of Pro was released from microparticle blends prepared by W/O/W (Pro) and O/W (CBZ) methods with DTI of 60 min. It has to be emphasized that the Pro was inside of microparticle and CBZ was on outer surface of microparticle. Thus only the drug located close to the outer surface could be initially released. The release of surface associated drug creates water-filled channels that allow subsequent diffusion of the drugs located inside the microparticles. A major mechanism for release of Pro and CBZ are diffusion through water-filled pores.

**Figure 6:**
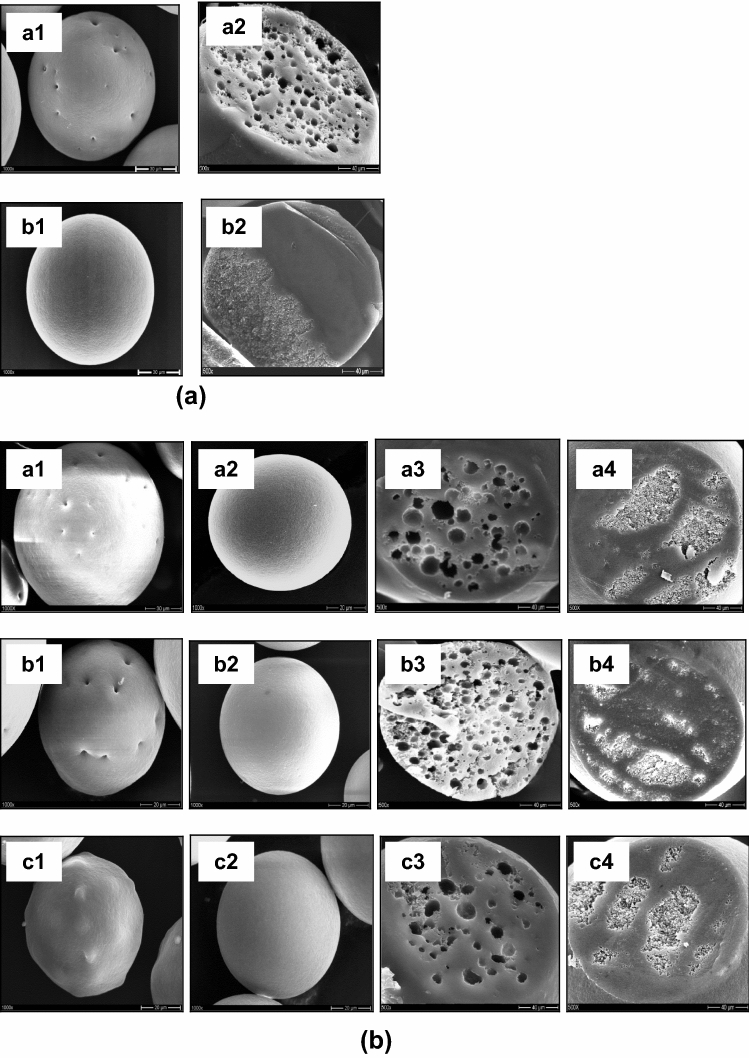
SEM pictures of ethyl cellulose microparticle blends with varying dispersion time interval between primary emulsion and primary oil phase (higher magnification and cross-section). (**a**) a.1–2. Pro (W/O/W) and b.1–2. CBZ (O/W), and (**b**) a.1–4. CBZ (O/W) and Pro (W/O/W), DTI: 60 min, b. Pro (W/O/W) and CBZ (O/W), DTI: 0 min and c.1–4. Pro (W/O/W) and CBZ (O/W), DTI: 60 min.

Based on release data for each microparticle blends, it can be assumed that there is interaction between first primary emulsion (Pro) and second primary oil phase (CBZ) during preparation process of microparticle blends. The surface morphology of microparticles blends (Fig. 2) and FBRM data on particle size mean before and after addition of second primary oil phase into single external aqueous phase (Fig. 3) have suggested it. Furthermore, the microparticles' cross sections revealed a porous inner structure and the absence of pores (Fig. 6).

The internal structure of CBZ loaded EC microparticles appeared to be dense with absence of pores irrespective of the preparation technique (O/W method) (Fig. 6a.b2). In EC microparticles containing Pro produced by the W/O/W technique, a very porous interior structure was discovered (Fig. 6a.a2). The observed absence of pores in the present O/W method is of major importance for the underlying drug release mechanisms, because drug diffusion through water-filled cavities is much faster than through dense polymeric networks. For microparticle blends which were prepared with DTI of 60 min the internal structure appeared reducing in the number of pores and size of pores (Fig. 6b.c3).

This behavior might be explained by the interaction of the second primary oil phase (CBZ) with hard particles from the first primary emulsion (Pro), in which the second primary oil phase (CBZ) occluded and coated pores on the hard particle's surface (Fig. 6b.c1). Optical microscopy images back up this hypothesis. It means that the first W/O/W (Pro) emulsification stage and the second (CBZ/dye) emulsification stage produced two types of microparticle blends (Fig. 2.c1). A microparticle with a black plaque on the surface and black microparticles was shown in this image.

Figures 1 and 6 shows the surface structure of the microparticles and microparticles blends prepared with the five kinds of methods. It can be seen that their surface morphology differs significantly. The Pro loaded microparticles (W/O/W) possessed several porous surface and had a sponge-like porous structure. However, a smoother and nonporous surface was formed on CBZ loaded microparticles (O/W). Highly porous microparticles were prepared by a double emulsion (W/O/W). It was caused by the inner aqueous phase. The aqueous droplets are precursors of pores and are the result of phase separation occurring in the organic phase during the hardening of the microparticles^2–5^. The microparticles blends prepared with DTI of 0 min (Pro (W/O/W) and CBZ (O/W)) and DTI of 60 min (CBZ (O/W) and Pro (W/O/W)) had a smoother, denser and porous surface compared with the microparticles blends with DTI of 60 min (Pro (W/O/W) and CBZ (O/W)) had a rough and non porous surface. Evidently, the morphology of the microparticles blends was affected by the DTI and type of first emulsion. This phenomenon was due to the difference in method between the microparticles blends. The microparticles blends prepared from a method with DTI of 60 min (Pro (W/O/W) and CBZ (O/W)) had produced two population of microparticles more quickly during the preparation process. The microparticle blends consist of blocked or coated miroparticles (Pro) and microparticles (CBZ). The reason for the porous surface of Pro loaded microparticles (W/O/W), was that the solvent had evaporated too quickly from the microparticles. Vaporization of the solvent inside the microspheres will cause disruption of the polymer film.

In order to better understand the surface properties of the obtained EC polymer and, as a result, emulsion characteristics which foreshadow the size and porosity of the final microparticle blends, SEM observations were carried out. These observations have been carried out on the intact microparticles to analyze their shape, roughness and surface porosity but also on some of their cross-sections in view to future detail their inner porosity. As seen in Figs. 1 and 6, SEM images clearly demonstrated that an introduction of the EC microparticle blends in the aqueous phase of the emulsion provided fabrication of spherical microparticles with smooth surface. Similar regular morphology has been observed for the EC microparticle blends prepared by adding the second emulsion with DTI of 0 min and DTI of 60 min in the external equeous phase of the emulsion. However, the obtained surface roughness was higher on the EC microparticle blends prepared with DTI of 60 min than with other samples.

SEM observations of the cross-sectioned samples showed a significant difference in internal structure of the EC microparticle blends prepared with DTI of 0 min and DTI of 60 min. The DTI of 60 min resulted in formation of the little and decrease porous inner microstructure while the microparticle surface remained regular, rough, smooth and non-porous. We suppose that smooth surface could be preferable for promoting cell adhesion at a first stage of cell adhesion, whereas the macroporous internal structure of the microspheres provided the benefits during the microcarrier degradation, i.e. larger surface area for cell anchorage and facilitated diffusion of nutrients and cell penetration. Thus, both approaches using the EC polymers for microparticle fabrication were successful in terms of the total microparticle yield, size distribution and surface morphology.

## Conclusion

Novel microparticle blends comprising Pro and CBZ have a lot of promise as controlled-release drug delivery systems. Pro release was slower than CBZ release in microparticle blends (with DTI of 60 min) than Pro release in microparticle blends (with DTI of 0 min) and microparticles normal. The particle size of microparticles from the first primary emulsion (Pro) was smaller than the particle size of microparticles after the addition of the second oil phase (CBZ) according to FBRM experiments (with DTI of 60 min). The release of Pro and CBZ enhanced as particle size decreased. Pro release was slower than CBZ release for all size fractions. The physical properties of microparticle blends were impacted by dispersion time interval and emulsification stage in the preparation process.

## Experimental section

### Microparticle preparation

#### Microparticle containing propranolol HCl or carbamazepine

Drug loaded microparticles based on EC were prepared using an oil-in-water (O/W) and a water-in-oil-in-water (W/O/W) solvent evaporation method. The drug loaded systems contained either one drug only (Pro or CBZ). For the O/W method, 300 mg of EC were dissolved in 3 ml dichloromethane. 43 mg CBZ were dissolved within this organic phase. The organic phase was then emulsified into 800 ml aqueous PVA solution (0.25% w/v) containing 0.5 M NaCl and NaOH at pH 12. The emulsion was stirred for 4 h at 500 rpm with a propeller stirrer (Heidolph Elektro GmbH & Co. KG, Kelheim, Germany) to allow microparticle hardening.

For the W/O/W method, 43 mg Pro were dissolved in 0.25 g purified deionized water. Pro aqueous solution was first emulsified by probe sonication (Sonoplus® HD 250, Bandelin Electronic GmbH & Co. KG, Berlin, Germany) for 30 s under ice-cooling into 3 ml dichloromethane containing 300 mg of EC. This first emulsion (W/O) was then dispersed into 800 ml aqueous PVA solution (0.25% w/v) containing 0.5 M NaCl and NaOH at pH 12. A W/O/W emulsion was formed by extensive stirring with a propeller stirrer for 4 h at 500 rpm to allow microparticle hardening. In all cases, after 4 h the microparticles were separated from the external aqueous phase by wet sieving (stainless steel test sieves ISO 3310–40, 70, 100 and 160 µm) followed by washing with 200 ml deionized water, desiccator-drying for 24 h and storage in a desiccator.

#### Microparticle blends containing propranolol HCl and carbamazepine

The first primary emulsion containing Pro (W/O/W) and second primary oil phase containing CBZ (O/W). For the W/O/W method, 43 mg Pro were dissolved in 0.25 g purified deionized water. Pro aqueous solution was first emulsified by probe sonication for 30 s under ice-cooling into 3 ml dichloromethane containing 300 mg of EC. This gave the first primary emulsion containing Pro. For the O/W method, 300 mg of EC were dissolved in 3 ml dichloromethane. 43 mg CBZ were then dissolved in this organic phase. This process produced the second primary oil phase containing CBZ. Following, the first primary emulsion containing Pro and the second primary oil phase containing CBZ were dispersed in an external aqueous phase (800 ml aqueous PVA solution [0.25% w/v] containing 0.5 M NaCl and NaOH at pH 12), with dispersion time intervals (DTI) of 0 and 60 min, and stirred for 4 h at 500 rpm with a propeller stirrer to allow microparticle hardening. The subsequent process steps were similar to the preparation of microparticle containing single drug process.

#### Determination of the actual drug loading and encapsulation efficiency

Microparticles (10 mg) were extracted in 1 ml methanol, followed by agitation in a horizontal shaker (IKA HS 501 digital horizontal Shaker, Janke & Kunkel GmbH & Co. KG IKA Labortechnik, Staufen, Germany) for 2 h (n = 3). 0.1 ml of methanol extract was diluted in 10 ml of pH 7.4 phosphate buffer. The polymer was separated from aqueous solution by filtration using filter paper (Whatman®, GE Healthcare UK Limited, Buckinghamshire, UK). Pro and/or CBZ concentration in the obtained aqueous solution was determined by UV-spectrophotometry at wavelengths of 289 nm and 285 nm, respectively (HP 8453 UV–Vis spectrophotometer, Agilent Technologies Deutschland GmbH, Waldbronn, Germany). The actual drug loading and encapsulation efficiency were calculated as follows:

1 $${\text{Actual drug loading }}\left( {{\% }} \right) = \left( {\text{drug mass in microparticles/mass of microparticles}} \right) \, \times \, 100 \, \%$$

2 $${\text{Encapsulation efficiency }}\left( {{\% }} \right) = \left( {\text{actual drug loading/theoretical drug loading}} \right) \, \times \, 100 \, \%$$

For microparticle blends, the amounts of incorporated Pro and CBZ were determined UV-spectrophotometrically by simultaneously measuring at wavelengths of 227 and 285 nm. The subsequent process steps were similar to the above process.

### Particle size analysis

Particle size mean and size distribution of the microparticles were measured by focused beam reflectance measurement. FBRM probe (FBRM D600T, Mettler Toledo AutoChem, Inc., Redmond, WA, USA) was immersed and positioned in the emulsification vessel (WO/W and O/W emulsions mentioned above) to ensure good flow against the probe window and hence allowing a representative sample of the particle system to be measured. The measurement range of the FBRM D600T probe is 0.25–4000 μm. In these experiments, FBRM measurements were performed every 10 s, during a period of 4 h. All batches were measured in triplicate. The size information was extracted through the iC FBRM 4.0 software (Mettler Toledo AutoChem, Inc., Redmond, WA, USA).

### Microparticle characterization

#### Optical microscopy

Microparticles were spread on microscope slides and observed with an optical light microscope (Axiotrop 50, Carl Zeiss AG, Jena, Germany) equipped with an image analysis system (INTEQ Informationstechnik GmbH, Berlin, Germany) consisting of a digital camera (type MC1) and the software (version 1.4.1).

#### Scanning electron microscopy

The external and internal morphology of microparticles was analysed by scanning electron microscopy (SEM). For surface imaging, the microparticles were fixed on a sample holder with double-sided tape. To investigate the inner structure, the particles were spread on transparent tape and then cut with a razor blade. All samples were coated under argon atmosphere with gold to a thickness of 8 nm in a high-vacuum (SCD 040, Bal-Tec GmbH, Witten, Germany). Samples were then analysed on the scanning electron microscope (S-4000, Hitachi High-Technologies Europe GmbH, Krefeld, Germany).

#### In vitro drug release studies

10 mg microparticles/microparticle blends (particle size: < 70 µm) were placed in 10 ml pH 7.4 phosphate buffer (USP XXIV) and shaken at 37 °C in a horizontal shaker (GFL 3033, Gesellschaft für Labortechnik GmbH, Burgwedel, Germany) at 75 rpm. At predetermined time points, 1 ml samples were withdrawn and replaced with 1 ml fresh medium each 7 days, filtered and analyzed. Pro and/or CBZ concentration was detected UV spectrophotometrically at wavelengths of 289 nm and 285 nm, respectively (n = 3) (HP 8453 UV–Vis spectrophotometer, Agilent Technologies Deutschland GmbH, Waldbronn, Germany).

For microparticle blends, the concentration of Pro and CBZ were determined UV-spectrophotometrically by simultaneously measuring at wavelengths of 227 and 285 nm (n = 3).
